# Impaired motor unit profile in Fabry disease: A quantitative electrophysiological study

**DOI:** 10.1016/j.cnp.2026.08.010

**Published:** 2026-08-25

**Authors:** Aysegul Gunduz, Aysegul Akkan Suzan, Mevlüt Tamer Dinçer, Şeyda Gül Özcan, Nurhan Seyahi

**Affiliations:** a Istanbul University-Cerrahpasa, Cerrahpasa Medical Faculty, Department of Neurology, Istanbul, Türkiye; b Istanbul University-Cerrahpasa, Cerrahpasa Medical Faculty, Department of Internal Medicine, Division of Nephrology, Istanbul, Türkiye

**Keywords:** Fabry disease, Motor unit number index (MUNIX), Motor unit size index (MUSIX), Compound muscle action potential (CMAP), Small-fiber neuropathy

## Abstract

**Objective:**

To investigate the relationship between routine electrophysiological findings, sympathetic function, and the number and size of functioning motor units in Fabry disease.

**Methods:**

Thirteen patients with Fabry disease and thirteen healthy individuals were evaluated. Nerve conduction studies and sympathetic skin responses were recorded from upper and lower limbs. Motor unit function was analyzed using the Motor Unit Number Index (MUNIX) technique by recording the first dorsal interosseous (FDI) muscle following ulnar nerve stimulation and the tibialis anterior (TA) muscle following peroneal nerve stimulation.

**Results:**

The MUNIX protocol demonstrated excellent reproducibility. MUNIX had excellent reproducibility (coefficient of variation: 20.7% in FDI; 14.7% in TA). FDI-MUNIX and MUSIX values were comparable between groups, whereas TA-CMAP amplitude and MUSIX were significantly lower in patients than in healthy controls, with no significant difference in TA-MUNIX. Correlation analysis revealed a positive association between Lyso-Gb3 and MUNIX-FDI (ρ = 0.55, *p* = 0.049) and inverse correlations between neuropathy scores and MUSIX-TA (ρ = −0.72, *p* = 0.013; ρ = −0.76, *p* = 0.004).

**Conclusions:**

The observed reductions in CMAP and MUSIX, particularly in the TA, indicate subtle length-dependent motor unit involvement despite preserved strength and normal MUNIX values.

**Significance:**

This study provides the first evidence that MUNIX can detect early or subclinical motor unit alterations in Fabry disease.

## Highlights

• Motor Unit Number Index was applied for the first time in Fabry disease.
• Subtle distal motor unit alterations are present despite preserved strength.
• Globotriaosylsphingosine correlated with upper extremity motor neuron profile.

## 1 Introduction

Fabry disease is an X-linked multisystemic disorder that affects several organs, including the skin, eyes, kidneys, heart, brain, and peripheral nervous system (Chan and Adam, 2018). The disease results from a deficiency of the α-galactosidase A (α-Gal A) enzyme, leading to the accumulation of globotriosylceramide (GL3) in vascular endothelial and smooth muscle cells. This progressive lipid accumulation causes vascular occlusion, ischemia, and organ dysfunction. Classical Fabry disease develops before teens when α-Gal A enzyme activity is below 1% (Chan and Adam, 2018). In the peripheral nervous system, GL3 deposits in the autonomic and dorsal root ganglia disrupt the function of voltage-gated ion channels and small nerve fibers (Michaud et al., 2020). Symptoms that begin in childhood include periods of discomfort, periodic crises of severe pain in the extremities (acroparesthesia), angiokeratomas, sweating abnormalities (hypohidrosis, anhidrosis, rarely hyperhidrosis), corneal opacity, digestive tract problems such as abdominal pain, nausea, or diarrhea, tinnitus, and hearing loss (Chan and Adam, 2018). As the condition progresses without treatment, it eventually results in kidney damage, cardiac arrhythmia, unexplained left ventricular failure, and unexplained stroke.

Although enzyme replacement therapy (ERT) can effectively reduce endothelial GL3 accumulation, patients may continue to experience small-fiber neuropathy and pain, suggesting that ischemia and fibroblast alterations may persist despite treatment (Mayes et al., 1982; Hasholt et al., 1988). Indeed, while nerve conduction studies in patients with Fabry disease often yield normal results (Boluk et al., 2022; Scott et al., 1999), an interesting study investigating multiple nerve excitability properties has demonstrated that poor nerve perfusion contributes to axonal damage (Tan et al., 2005). Furthermore, recent electrophysiological studies have demonstrated evidence of motor nerve involvement in a subset of patients (Koszewicz et al., 2026). These findings indicate that subclinical motor unit impairment may occur in Fabry disease, potentially related to chronic ischemia or tissue remodeling.

Considering these mechanisms, a more detailed assessment of motor function is warranted. The Motor Unit Number Index (MUNIX) is a quantitative electromyographic technique developed by Nandedkar et al. (2010), providing an index directly correlated with the number of motor units. For a given muscle, MUNIX requires the compound muscle action potential (CMAP) and various levels of voluntary muscle contractions recorded as surface interference patterns (SIP). Using a mathematical model that integrates CMAP and SIP data, a “motor unit count” can be derived (8,9). MUNIX has shown a strong correlation with other motor unit number estimation (MUNE) techniques and demonstrates minimal inter- and intra-observer variability in both patients with amyotrophic lateral sclerosis (ALS) and healthy individuals (Nandedkar et al., 2010; Neuwirth et al., 2015).

Given that Fabry disease is classically considered a sensory–autonomic neuropathy with preserved motor function, MUNIX analysis provides an opportunity to objectively confirm motor unit integrity and detect possible early or subclinical changes that may not be evident in conventional electrophysiological studies, since metabolic or ischemic factors may also subtly influence motor unit physiology. Therefore, the aim of this study was to evaluate motor unit integrity using MUNIX analysis in Fabry disease and to explore its relationship with clinical and electrophysiological findings.

## 2 Patients and methods

This is a cross-sectional cohort study conducted in the Neurology and Nephrology departments of our center. We enrolled all consecutive adult patients (aged>18 years) with Fabry disease who were admitted to a tertiary care university hospital between April and July 2024, as well as healthy individuals of similar age and sex for comparison.

### 2.1 Patients

The diagnosis of Fabry disease was established based on typical findings, including a positive family history, decreased plasma α-Gal A enzyme activity, and confirmation through genotype testing. The pathogenic mutation was confirmed in all patients included in the study. The exclusion criteria for the patient group were severe renal failure in Fabry disease that may cause large fiber neuropathy, another disease that may cause neuropathy (advanced organ failure, malignancy, HIV infection, rheumatological diseases, steroid use, etc.), and, for both groups, a disease that would prevent or interfere with the electrophysiological examinations and no consent to participate in the evaluation.

The local ethics committee approved the study; the patient and control groups were informed about the examinations, and we obtained the necessary consent.

## 3 Method

### 3.1 Clinical evaluation

The assessment included age, gender, a comprehensive clinical history, medication use, and an extensive neurological examination. Demographic, clinical, neurologic, and laboratory findings for the patients were obtained from their records. Hearing loss, hypertension, skin, central nervous system, and cardiovascular or renal involvement were noted. The neurological findings were evaluated using the Leeds Assessment of Neuropathic Symptoms and Signs (LANSS) pain scale (Bennett, 2001; Koc and Erdemoglu, 2010) and the modified Toronto Clinical Neuropathy Score (mTCNS) (Bril et al., 2009). We retrieved information regarding cardiac and renal involvement, as well as plasma lyso-globotriaosylsphingosine (Lyso-Gb3) levels, from the electronic patient files.

• *Leeds Assessment of Neuropathic Symptoms and Signs (LANSS) pain scale:* This simple bedside test analyzes and classifies pain. It has two parts: a patient-completed questionnaire and a brief clinical assessment. The purpose of the test is to help clinicians assess the severity of the pain or its causes. However, it is also the only published tool with validity for discriminating between neuropathic and nociceptive pain, regardless of the disease-based diagnostic methods. According to various research reports, these LANSS tests correctly classified the patients as suffering from nociceptive and neuropathic pain in four out of every five patients experiencing chronic pain (Bennett, 2001; Koc and Erdemoglu, 2010).
  It has a 7-item pain scale, comprising five symptom-related items and two examination items. The total score is 24. A patient with a score of 12 or more on this scale is diagnosed as suffering from neuropathic pain to some degree.
• *‘Modified Toronto Clinical Neuropathy Score System’ (mTCNS):* This is a brief, semi-structured clinical interview and examination method. The TCNS was first designed to detect early diabetic sensorimotor neuropathy and is weighted to emphasize sensory symptoms and deficits, which are the typical first symptoms and signs of diabetic neuropathy (Bril and Perkins, 2002). mTCNS was derived from TCNS; it includes sensory tests and excludes reflex testing. The mTCNS is a validated scale for diabetic sensorimotor neuropathy that correlates with its severity (Bril et al., 2009). It is a simplified method for using the neurological examination to assess peripheral sensory perception and the presence of neuropathic symptoms. The mTCNS enhances the reliability of the scale by eliminating reflex tests, which are variable between raters, and increases sensitivity compared to the TCNS, as the loss of reflexes occurs later in the neuropathy process (Bril et al., 2009). Both scales are valid instruments for detecting the presence and severity of neuropathy.
  The cut-off value for diagnosing polyneuropathy was ≥3 (sensitivity, 98.4%; specificity, 85.7%; positive likelihood ratio, 6.88) (Idiaquez et al., 2023).

### 3.2 Electrophysiological examinations

Electrophysiological examinations were conducted on the right upper and lower extremities using an EMG device (Cadwell Sierra Summit). If any abnormal findings were detected, we recorded both the upper and lower extremities. During the tests, the skin temperature was maintained at 32 °C for the upper limbs and 30 °C for the lower limbs. Nerve conduction studies were performed in all participants, and needle electromyography was conducted in selected cases as indicated.

• *Nerve conduction studies and needle electromyography:* We performed nerve conduction studies and EMG using conventional methods as described in the literature (Kimura, 2006) to classify patients as abnormal or normal based on the findings of nerve conduction studies and exclude patients with abnormal findings.
  Motor and sensory conduction studies were performed on the following nerves:
• Upper limbs: median and ulnar nerves (motor and sensory branches).
• Lower limbs: peroneal, tibial, and sural nerves.

A total of 20 F-waves were recorded on the APB muscle after supramaximal stimulation.

The following parameters were measured:

• baseline-to-peak amplitude, onset latency, and conduction velocity of motor responses,
• minimum F-wave latency and persistence,
• onset latency and peak-to-peak amplitude of sensory responses.

For needle EMG, the presence of fibrillation potentials, positive sharp waves, and fasciculations was noted. Interference patterns and motor unit potentials were analyzed semi-quantitatively to assess motor unit morphology and recruitment characteristics.

• Sympathetic Skin Response (SSR) Recording: Sympathetic skin response (SSR) was recorded to assess sudomotor autonomic function as a reflection of small fibers in all participants (Kimura, 2006). Recordings were performed in a quiet, temperature-controlled room with ambient temperature maintained between 24 °C and 26 °C to ensure stable sympathetic activity. Skin temperature was monitored throughout the procedure and kept above 32 °C at the recording sites.
  SSR was elicited using electrical stimulation of the median nerve at the wrist for the upper limb and the tibial nerve at the ankle for the lower limb. Each stimulation consisted of a single square-wave pulse of 0.2 ms duration and current intensity of 40 mA, delivered at irregular intervals (10–20 s apart) to minimize habituation. Surface recording electrodes (Ag/AgCl disposable type) were placed on the palmar surface of the hand and the plantar surface of the foot, with the reference electrodes placed on the dorsum of the same limb. A ground electrode was attached to the forearm or lower leg, respectively. SSR signals were recorded using the Cadwell Sierra Summit EMG system, with band-pass filters set at 0.5–2000 Hz and a sweep speed of 500 ms/division.
  A total of five reproducible SSR responses were obtained for each limb, and the mean latency and amplitude values were calculated from at least three consistent responses.
  The following parameters were measured:
• The onset latency (ms) was measured from the onset of the stimulus artifact to the beginning of the first deflection of the SSR waveform.
• The amplitude (mV) was measured from the negative to the positive peak of the response.

Absence of SSR after repeated stimulation was interpreted as sympathetic dysfunction.

SSR recordings were considered normal when reproducible responses were obtained bilaterally within the expected latency range, as defined by laboratory norms.

**Fig. 1:**
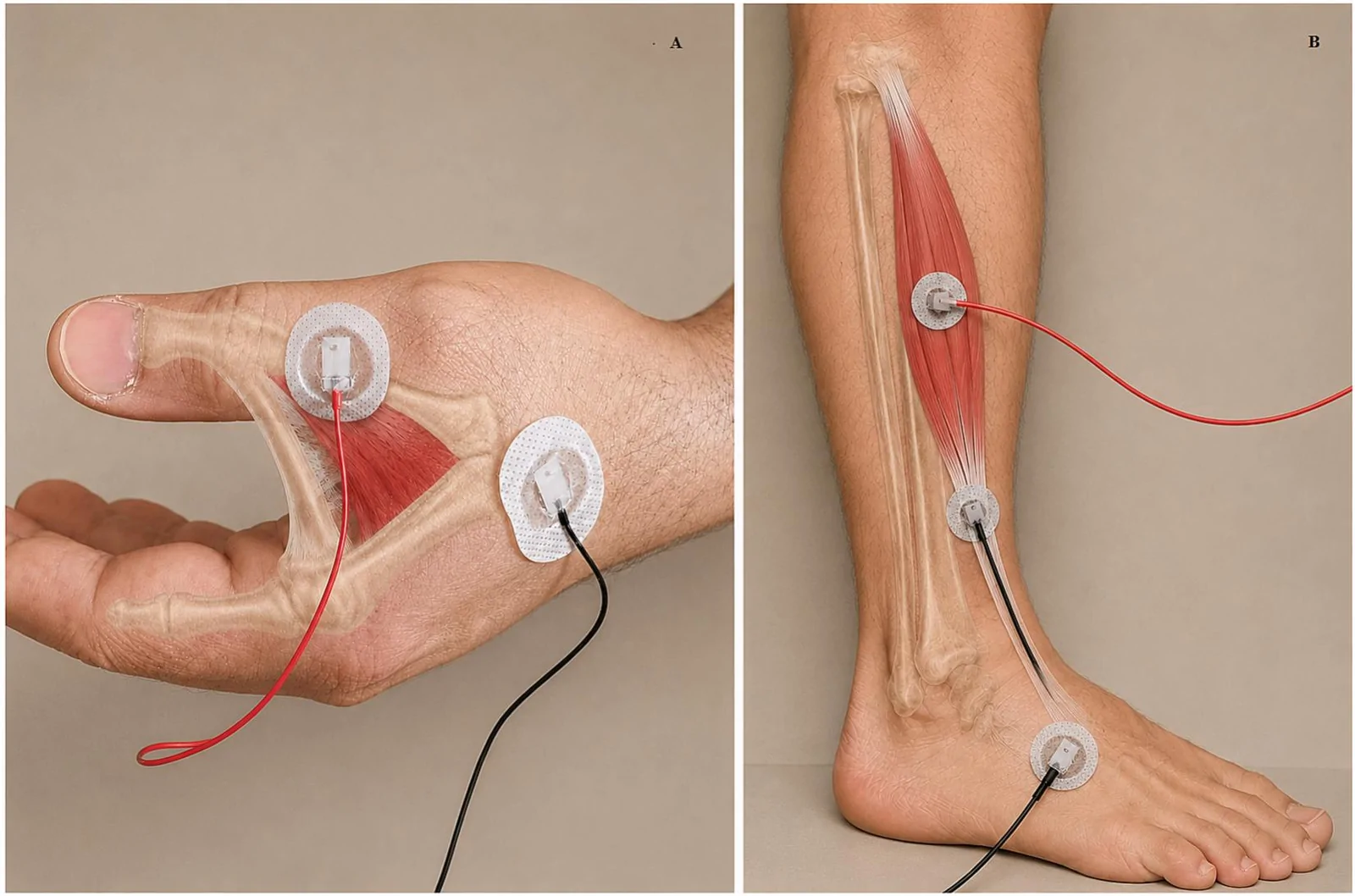
Electrode placement for motor unit number index (MUNIX) recordings. (A) First dorsal interosseous (FDI) muscle. The active recording electrode was positioned over the motor point of the FDI muscle within the first web space between the first and second metacarpals, while the reference electrode was placed over the proximal phalanx of the index finger. (B) Tibialis anterior (TA) muscle. The active recording electrode was positioned over the motor point of the TA muscle, approximately at the proximal one-third of the line connecting the fibular head and the medial malleolus, and the reference electrode was placed over the distal tendon of the TA muscle. In both panels, semi-transparent anatomical overlays illustrate the underlying muscles and bony landmarks relative to the electrode positions. The ground electrode was placed on the dorsum of the hand for FDI recordings and on the dorsum of the foot for TA recordings.
  Offline, CMAP and SIP data were analyzed using a dedicated program. The signal quality was visually inspected to exclude traces contaminated by noise, tremor, or movement artifacts. From the recorded SIP epochs, 300-ms segments of stable activity were selected for further analysis. For each SIP, the area and power were calculated, followed by computation of the ideal case motor unit count (ICMUC) according to the method of Nandedkar et al. (2004).
  The negative phase of the maximal CMAP was used to determine CMAP peak amplitude, area, and power. To minimize the influence of volume-conducted activity from neighboring muscles, only signals that fulfilled the following criteria were included in the analysis:
• SIP area > 20 μV·ms,
• ICMUC <100, and
• SIP area / CMAP area > 1.

• *Motor unit number index (MUNIX) protocol:* MUNIX measurements were performed following the procedure described by Nandedkar et al. (2010) and further standardized by Nandedkar et al. (2018). Recordings were obtained from the right first dorsal interosseous (FDI) and tibialis anterior (TA) muscles using disposable surface disk electrodes and a hand-held bipolar stimulator. All examinations were performed by a single experienced examiner (A.A.S.). Filter settings were set between 3 Hz and 10 kHz, and the stimulation pulse width was 0.2 ms.
  Fig. 1 shows the electrode placement and setup. First, surface EMG interference patterns (SIP) were recorded during voluntary muscle contractions against manual resistance provided by the operator. Two series of SIP recordings were made to obtain a wide dynamic range of activity. Two series of SIP recordings were performed at 8–10 incrementally increasing force levels, ranging from minimal to maximal effort. For each contraction, the participant was instructed to maintain a steady force for approximately 2–3 s while the operator ensured signal stability.

Using power regression analysis on the valid ICMUC-SIP-area pairs (as defined in Nandedkar et al., 2018), the MUNIX value for each muscle was calculated. At least 8 out of 10 valid combinations were required to obtain a reliable regression. Finally, the motor unit size index (MUSIX) was derived according to the formula:

MUSIX = (CMAP amplitude x 1000) \ MUNIX.where CMAP amplitude is expressed in millivolts (mV) and MUSIX in microvolts (μV).

To assess intra-rater reproducibility, the MUNIX procedure was repeated twice under identical conditions in all healthy controls and in 10 of the 13 patients with Fabry disease.

### 3.3 Statistical analysis

We first analyzed normality using a Shapiro-Wilk test. Because of the relatively small sample size, effect sizes were additionally reported as Hedges' *g* for the primary between-group comparisons. Between-group comparisons (patients vs. healthy individuals) were performed using the Mann–Whitney *U* test for non-normally distributed data and the independent-samples *t*-test for normally distributed data.

The Wilcoxon signed-rank test was used to compare the first and second recordings obtained from all participants included in the test–retest analysis, in order to evaluate measurement reproducibility. Correlation analyses were performed to examine the relationships between clinical scale scores and SSR parameters, as well as CMAP, MUNIX, and MUSIX values.

All statistical analyses were conducted using SPSS version 20.0 (IBM Corp., Armonk, NY, USA). A *p*-value ≤0.05 was considered statistically significant. To account for multiple comparisons of the primary electrophysiological outcome measures, Bonferroni correction was applied. For the six between-group comparisons (FDI-CMAP, FDI-MUNIX, FDI-MUSIX, TA-CMAP, TA-MUNIX, and TA-MUSIX), statistical significance was defined as a two-sided *p-*value <0.0083.

## 4 Results

During the study period, 16 patients with Fabry disease and 13 age- and sex-matched healthy individuals were evaluated (Table 1). Three patients were excluded due to severe renal failure accompanied by large-fiber neuropathy.

**Table 1:** Clinical findings of participants with and without Fabry disease.

	Fabry disease *n* = 13	Healthy individuals n = 13	P
Age, years	38.5 ± 13.1 (25–63)	38.2 ± 14.9 (19–65)	0.960^a^
Sex, M n (%)	3 (23.1)	3 (23.1)	1.0
LANSS	6.9 ± 2.8 (0–16)	3.1 ± 2.6 (0–5)	0.034*^,b^
mTCNS	9.1 ± 6.7 (0−23)	1.2 ± 0.7 (0–2)	<0.001*^,b^
Plantar SSR amp, μV	177.6 ± 240.5 (0–839)	648.3 ± 383.0 (264–1276)	0.007*^,b^
Plantar SSR lat, ms	1773.6 ± 416.4 (1200–2507)	1338.3 ± 342.1 (906–1898)	0.059 ^a^
Palmar SSR amp, μV	686.1 ± 645.7 (0–1988)	1283.3 ± 1152.5 (582–4254)	0.115^b^
Palmar SSR lat, ms	1068.8 ± 466.7 (796–1500)	1043.0 ± 360.3 (758–1695)	0.894^b^

Numeric values are presented as mean ± SD (min + max); a, *t*-test for independent groups; b, Mann-Whitney *U* test.

*p < 0.05, ** < 0.005.

### 4.1 Clinical findings

Patients with Fabry disease predominantly reported pain, numbness, and burning; none exhibited objective muscle weakness. All except one patient were receiving enzyme replacement therapy at the time of examination (Table 2). One patient had been recently diagnosed and was treatment-naïve. On neurological examination, six patients (37.5%) demonstrated reduced deep-tendon reflexes and distal sensory loss. Patients exhibited significantly higher LANSS and mTCNS scores than healthy individuals (LANSS: 6.9 ± 2.8 vs 3.1 ± 2.6, *p* < 0.034; mTCNS: 9.1 ± 6.7 vs 1.2 ± 0.7, *p* < 0.001). None of the healthy individuals had abnormal findings on clinical or neurological examination.

**Table 2:** Clinical characteristics of the patients with Fabry disease.

Patient	Age	Gender	Mutation	Phenotype	ERT	Lyso-Gb3 ng/mL	Renal involvement (1 = yes, 0 = no)	Cardiac involvement (1 = yes, 0 = no)
Patient 1	32	F	p.R301Q	Other	agalsidase alfa	1.4	0	1
Patient 2	51	F	p.R301Q	Other	agalsidase alfa	1.7	1	1
Patient 3	41	M	p.K240Efs X9	Classic	agalsidase alfa	37.1	1	1
Patient 4	52	F	p.L268F	Other	agalsidase beta	4.1	1	1
Patient 5	20	M	p.D266N	Classic	agalsidase beta	10.1	0	0
Patient 6	47	F	p.N34H	Other	agalsidase alfa	7.3	0	0
Patient 7	48	F	p.R227*	Classic	agalsidase alfa	10.7	1	1
Patient 8	25	F	p.D266N	Classic	agalsidase alfa	1.5	0	0
Patient 9	25	M	p.L268	Classic	agalsidase alfa	41.6	1	1
Patient 10	27	F	p.G373R	Other	agalsidase alfa	3	0	1
Patient 11	30	F	p.G373R	Other	agalsidase alfa	2.8	0	0
Patient 12	40	F	p.G373R	Other	agalsidase alfa	2.6	0	1
Patient 13	63	F	p.I270T	Late onset	none	1.1	0	1

ERT, Enzyme Replacement Therapy; F: Female, M: Male, Lyso-Gb3, globotriaosylsphingosine; ng/mL, nanograms per milliliter.

### 4.2 Electrophysiological findings

All electrophysiological recordings were well tolerated and technically satisfactory. Three patients had sensory predominant length dependent axonal neuropathy, in whom needle EMG of the first dorsal interosseous, biceps brachii, tibialis anterior, and vastus medialis muscles demonstrated chronic neurogenic motor unit potential changes, supporting the presence of axonal motor involvement. Consequently, these patients were excluded from the final analysis, and the final study cohort consisted of 13 patients with Fabry disease. In the remaining cohort, two patients (12.5%) presented findings consistent with small-fiber neuropathy with clinical findings and absent SSR, and two (12.5%) had carpal tunnel syndrome, whereas one control subject (7.7%) had neurophysiological findings of carpal tunnel syndrome without overt symptoms (*p* = 0.540). Other findings were normal in these remaining patients.

### 4.3 Motor unit number index (MUNIX) and related parameters

Shapiro–Wilk testing demonstrated that all primary electrophysiological variables were normally distributed in both groups (*p* > 0.05). Therefore, between-group comparisons were performed using independent-samples *t*-tests. Effect size analysis demonstrated a negligible effect for TA MUNIX (Hedges' *g* = −0.08, 95% CI −0.88 to 0.72), a small effect for FDI MUNIX (−0.38, 95% CI −1.17 to 0.41), a moderate effect for FDI MUSIX (−0.71, 95% CI −1.52 to 0.10), and a large effect for TA MUSIX (−1.60, 95% CI −2.56 to −0.65), supporting the substantial reduction in TA MUSIX observed in patients with Fabry disease.

The MUNIX protocol was repeated twice to assess reproducibility; no significant differences were observed between the two recordings (*p* > 0.05, Wilcoxon signed-rank test). Quantitative comparisons of CMAP, MUNIX, and MUSIX values for both the FDI and TA muscles are summarized in Table 3.

**Table 3:** Comparison of CMAP, MUNIX, and MUSIX values recorded on FDI and TA muscles in patients with Fabry disease and healthy individuals.^a^, ^b^

Muscle	Parameter	Fabry disease (*n* = 13)	Healthy individuals (n = 13)	*p*-value
FDI	CMAP amplitude (mV)	12.7 ± 3.7 (5.8–19.0)	14.7 ± 3.1 (12.3–22.9)	0.156^a^
FDI	MUNIX	185.4 ± 42.8 (141–250)	202.8 ± 48.1 (148–292)	0.340^a^
	MUSIX	72.5 ± 16.9 (41–104)	86.1 ± 22.4 (61–132)	0.125^b^
TA	CMAP amplitude (mV)	4.9 ± 0.6 (3.9–5.8)	6.6 ± 1.5 (4.4–8.3)	< 0.001**
	MUNIX	86.8 ± 16.2 (63–116)	88.5 ± 26.9 (55–155)	0.844^a^
	MUSIX	59.1 ± 8.2 (48–73)	85.7 ± 20.9 (52–115)	< 0.001**,^b^

Data are presented as mean ± SD. *P*-values were obtained using the independent-samples *t*-test. Effect sizes were estimated using Hedges' *g* with 95% confidence intervals (CI). Hedges' *g* (95% CI) for the primary electrophysiological outcomes was: FDI MUNIX: −0.38 (−1.17 to 0.41); FDI MUSIX: −0.71 (−1.52 to 0.10); TA MUNIX: −0.08 (−0.88 to 0.72); TA MUSIX: −1.60 (−2.56 to −0.65).

Abbreviations: CMAP = compound muscle action potential; MUNIX = motor unit number index; MUSIX = motor unit size index; FDI = first dorsal interosseous; TA = tibialis anterior.

* *p* < 0.05; ** *p* < 0.001.

a Independent-samples *t*-test.

b Mann–Whitney *U* test.

For the TA muscle, Pearson correlation coefficients were 0.922 and 0.843 for MUNIX and MUSIX, respectively, with coefficients of variation of 14.7% and 13.3%, respectively. For the FDI muscle, Pearson correlation coefficients were 0.894 and 0.912 for MUNIX and MUSIX, respectively, with coefficients of variation of 20.7% and 16.4%, respectively, while Bland–Altman analysis demonstrated low measurement bias and acceptable limits of agreement for both MUNIX and MUSIX measurements (Suppl Table 1).

In the FDI muscle, the mean CMAP amplitude was 12.7 ± 3.7 mV in patients with Fabry disease and 14.7 ± 3.1 mV in healthy individuals (*p* = 0.156). The mean MUNIX values were also comparable between the two groups (185.4 ± 42.8 vs 202.8 ± 48.1, *p* = 0.340).

Similarly, the MUSIX of the FDI did not differ significantly (72.5 ± 16.9 vs 86.1 ± 22.4, *p* = 0.125).

In contrast, significant differences emerged in the TA muscle. The CMAP amplitude was significantly lower in patients with Fabry disease (4.9 ± 0.6 mV) compared with healthy individuals (6.6 ± 1.5 mV, *p* < 0.001). Notably, all TA-CMAP amplitudes in both groups remained within the normal reference range of our laboratory (above 4 mV). Furthermore, although MUNIX values for the TA muscle were slightly lower in patients (86.8 ± 16.2) than in controls (88.5 ± 26.9), this difference did not reach statistical significance (*p* = 0.844). The MUSIX, calculated as the CMAP/MUNIX ratio, was significantly lower in the patient group (59.1 ± 8.2 vs. 85.7 ± 20.9, *p* < 0.001).

The CMAP amplitude of the TA muscle was significantly lower in patients with Fabry disease than in healthy controls (*p* < 0.001), and this difference remained statistically significant after Bonferroni correction. Likewise, the lower TA MUSIX values observed in the patient group (*p* < 0.001) remained significant after correction for multiple comparisons.

**Fig. 2:**
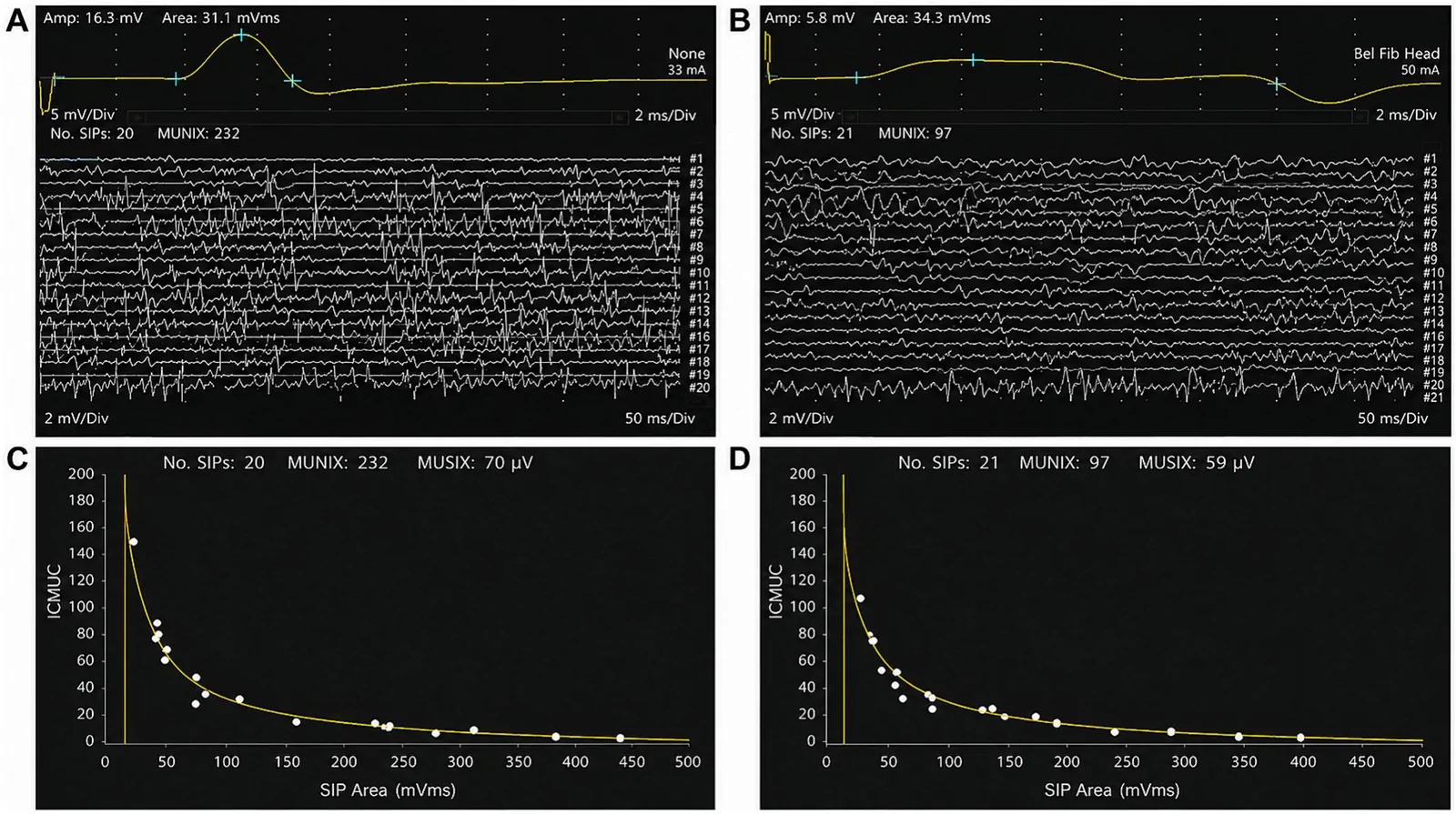
Representative MUNIX recordings from the first dorsal interosseous muscle (left column) and tibialis anterior (right column) muscles in a patient with Fabry disease. The upper panels show compound muscle action potential (CMAP) recordings; the middle panels display representative surface interference pattern (SIP) recordings obtained at different levels of voluntary contraction; and the lower panels present the corresponding regression plots used for MUNIX calculation. These examples illustrate the electrophysiological characteristics associated with reduced MUSIX in Fabry disease.

Fig. 2 demonstrates representative examples of MUNIX studies in a patient with Fabry disease.

### 4.4 Correlation analysis

Spearman's rank correlation analysis was performed to explore relationships among biochemical, clinical, autonomic, and electrophysiological parameters (Supp. Table 2 and Suppl Figure).

Clinical neuropathy scores were strongly interrelated: LANSS and mTCNS showed a positive correlation (ρ = 0.45, *p* = 0.19). Both LANSS (ρ = −0.72, *p* = 0.013) and mTCNS (ρ = −0.76, *p* = 0.004) showed significant inverse correlations with TA MUSIX, suggesting a potential association between neuropathic symptom severity and distal motor unit characteristics. In the autonomic domain, LANSS showed a negative correlation with plantar SSR amplitude (ρ = −0.75, *p* = 0.084), suggesting reduced sympathetic reactivity in patients with higher pain intensity.

Lyso-Gb3 levels showed a moderate positive correlation with plantar SSR amplitude (ρ = 0.67, *p* = 0.071) and a significant correlation with MUNIX of the FDI muscle (ρ = 0.55, *p* = 0.049), suggesting that higher substrate accumulation is associated with increased motor unit indices and altered autonomic responses. No significant association was observed between Lyso-Gb3 and MUSIX or CMAP of the TA muscle.

The supplementary table 2 summarizes the relationships between biochemical marker levels (Lyso-Gb3), clinical neuropathy and pain scores (LANSS, mTCNS), autonomic measures (palmar and plantar SSR amplitudes), and motor electrophysiological indices (MUNIX, MUSIX, and CMAP) obtained from the FDI and TA muscles.

## 5 Discussion

The key findings of this study were as follows: (i) the CMAP amplitude and MUSIX of the TA muscle after peroneal nerve stimulation were significantly lower in patients with Fabry disease than in healthy controls; (ii) no significant difference was observed in the FDI muscle; and (iii) significant positive correlations were observed between Lyso-Gb3 and MUNIX-FDI, whereas inverse correlations were found between LANSS and mTCNS scores and MUSIX-TA.

These findings suggest that motor unit integrity in the upper limb was largely preserved among patients receiving enzyme replacement therapy. However, upper limb motor involvement cannot be excluded, as previous studies have reported abnormalities in motor axonal excitability in the APB muscle (Tan et al., 2005). Although overt motor axonal loss is uncommon, subtle subclinical changes in motor unit function may occur in patients with Fabry disease, particularly in the lower limb muscles. The decline in MUSIX likely reflects the mathematical dependence of this index on CMAP amplitude rather than an independent physiological effect on motor unit size. Reduced MUSIX-TA values were associated with greater pain and neuropathy severity, indicating a relationship between clinical disease burden and lower-limb electrophysiological abnormalities. Although this pattern may be compatible with alterations in motor unit characteristics, MUSIX is a derived index and does not independently demonstrate changes in motor unit morphology or size.

When a motor unit is lost due to damage to its neuron or axon, the remaining muscle fibers may receive collateral innervation from surviving motor units. As a result, the total number of motor units decreases, but the CMAP may remain within normal limits. Under these conditions, the MUNIX is considered more sensitive than CMAP amplitude for detecting motor unit loss (Nandedkar et al., 2010). MUNIX values are typically reduced in conditions involving motor neuron degeneration, such as ALS, in which most muscles with low MUNIX values also show reduced CMAP amplitudes and muscle strength, as expected in advanced denervation. However, some muscles may present with reduced MUNIX despite normal CMAP and strength (Escorcio-Bezerra et al., 2016), suggesting subclinical changes in motor unit behavior.

In this study, patients with Fabry disease exhibited reduced CMAP and MUSIX values despite preserved MUNIX measurements. As all TA-CMAP amplitudes in both groups remained within the normal reference range, this indicates that the observed difference was statistically significant rather than a clinically overt electrophysiological abnormality. These findings suggest subtle changes in lower limb motor electrophysiology, although the physiological significance of the MUSIX reduction remains uncertain. Several mechanisms may underlie this pattern: (i) early neuropathic changes that affect motor unit efficiency or size without reducing their number; (ii) loss of larger motor units with compensatory recruitment of smaller ones, maintaining total count but lowering muscle response amplitude; and (iii) muscle plasticity and reorganization that preserve function despite reduced motor efficiency. Although Fabry disease has classically been regarded as a small-fiber neuropathy, recent evidence indicates that large motor fibers may also be affected. A recent case-control study demonstrated slowed motor conduction velocity in the ulnar and peroneal nerves, along with reduced peroneal CMAP amplitudes, particularly in patients with advanced disease and chronic kidney disease (Koszewicz et al., 2026). However, evidence from routine needle electromyography remains scarce, and most studies have reported normal or only subtle abnormalities in patients without clinically overt motor neuropathy. These findings suggest that motor involvement in Fabry disease may be subclinical and detectable only with more sensitive electrophysiological techniques. These interpretations remain speculative because muscle contraction force was not measured directly and objectively. Future studies combining MUNIX with quantitative force assessment or muscle imaging may better define these subclinical adaptations.

Fabry disease primarily affects sensory and autonomic fibers, resulting in burning pain, heat intolerance, and altered sweating due to degeneration of small myelinated Aδ and unmyelinated C fibers, while motor fibers are typically preserved. Previous studies using nerve conduction and quantitative sensory testing have consistently shown normal large-fiber and motor function, even in advanced stages (Boluk et al., 2022; Scott et al., 1999). The absence of significant MUNIX reduction in our cohort aligns with this pathophysiology. The mild decreases in CMAP and MUSIX—particularly in the distal TA—likely represent secondary or subclinical effects of vascular ischemia, metabolic stress, or mild muscle involvement rather than primary motor neuron pathology. Thus, Fabry disease remains essentially a sensory–autonomic neuropathy, with only minimal, length-dependent, and secondary motor involvement. Additionally, motor fiber involvement appears to emerge predominantly in the later stages of Fabry disease and tends to be more pronounced in patients with additional comorbid conditions, particularly chronic kidney disease (Koszewicz et al., 2026).

Lyso-globotriaosylsphingosine, , the deacylated derivative of globotriaosylceramide (Gb3), is a key biomarker of Fabry disease. Owing to deficient or reduced α-Gal A activity, Gb3 accumulates within lysosomes of vascular, smooth muscle, and neuronal cells, a fraction of which undergoes deacylation to form Lyso-Gb3. Elevated plasma Lyso-Gb3 correlates strongly with disease severity and the classical phenotype, serving as a sensitive indicator of treatment response. It also exhibits biological activity, promoting inflammation, oxidative stress, and small-fiber injury (Kuchar et al., 2024). A 30–70% reduction is typically expected after initiation of ERT or chaperone therapy; persistently high levels indicate suboptimal enzyme activity or advanced disease. In this study, most patients (≈77%) had mild or borderline elevations (<10 ng/mL), while three exhibited marked increases (10.1–41.6 ng/mL), reflecting heterogeneous disease activity. These biochemical results are consistent with predominantly sensory–autonomic involvement and minimal motor pathology, as supported by the electrophysiological findings. Pathophysiologically, Lyso-Gb3 contributes to peripheral nerve dysfunction through several mechanisms:

• Accumulation in dorsal root ganglia and small sensory fibers → leading to small-fiber neuropathy, pain, and impaired thermal sensation.
• Deposition in vascular endothelium → causing ischemia and impaired microcirculation.
• Proinflammatory effects → promoting cytokine release, oxidative stress, and fibrosis in neural and vascular tissues.

Our findings, on the other hand, correspond to mild-to-moderate systemic substrate accumulation and are consistent with predominantly sensory and autonomic involvement

whereas motor involvement is minimal, which aligns with our electrophysiological findings (normal MUNIX, mild CMAP/MUSIX changes). Thus, we suggest that with increasing Lyso-Gb3, there is a stronger association with small-fiber and autonomic dysfunction as shown by clinical scales, as well as possibly mild secondary muscle effects due to ischemia or metabolic stress, but no true motor neuropathy.

The interpretation of the motor unit findings warrants further clarification. Although MUNIX/MUSIX has been extensively validated as a quantitative biomarker of motor unit loss and is primarily used to monitor disease progression, its diagnostic role in peripheral neuropathies remains limited. Therefore, the reduced MUSIX values observed in our cohort should not be interpreted in isolation or regarded as definitive evidence of a length-dependent neuropathy. Rather, these findings should be considered complementary to the overall clinical and neurophysiological assessment. Reduced MUSIX values in the TA were associated with increasing neuropathic pain or clinical severity, suggesting smaller distal motor unit sizes in patients with more severe neuropathic manifestations. However, preservation of normal values in the FDI may instead suggest a distal or length-dependent vulnerability, which is more consistent with the known distribution of peripheral nerve involvement in Fabry disease. If the observed MUSIX reduction reflects secondary systemic mechanisms rather than a length-dependent neuropathic process, it remains unclear why comparable changes were not observed in the FDI muscle.

### 5.1 Study limitations

This study has several limitations. First, MUNIX measurements are sensitive to examiner technique and signal quality. To minimize variability, all recordings were performed by a single experienced examiner using a standardized protocol, resulting in high intra-rater repeatability. Correlation analyses were exploratory, and results should be interpreted cautiously. Second, the relatively small sample size reflects the rarity of Fabry disease, as well as our exclusion of patients with severe renal failure and large-fiber neuropathy; however, this selection process minimized confounding and potential bias. A further limitation, due to the relatively small sample size and the large number of correlation analyses performed, is an increased likelihood of Type I error. Consequently, these findings should be regarded as exploratory and hypothesis-generating, requiring confirmation in larger independent cohorts. Another factor that may influence the results is the stimulation protocol. Although standardized, the type and intensity of electrical stimulation can affect CMAP amplitude and, consequently, derived indices such as MUNIX. This limitation should be considered when comparing results across different studies or laboratories. Furthermore, SSR recordings were obtained using only electrical stimulation. Alternative stimulation modalities, such as auditory or inspiratory stimuli, were not applied; however, we paid attention to the potential influence of habituation of SSR responses, and we limited the number of applied stimuli. Finally, the absence of muscle force measurement limits the direct relationship between electrophysiological findings and functional outcomes.

## 6 Conclusion

In conclusion, patients with Fabry disease exhibited reduced TA-CMAP amplitude and MUSIX values, while MUNIX measurements remained preserved. These findings suggest subtle lower-limb motor electrophysiological changes; however, the significance of the observed MUSIX reduction remains uncertain and may be influenced by the lower CMAP amplitude. Although MUNIX did not differ significantly between groups, its combined interpretation with CMAP and MUSIX may provide additional insight into motor unit characteristics. MUNIX is a reproducible and efficient electrophysiological method that can be readily incorporated into standard EMG protocols. As one of the first studies to apply MUNIX in a Fabry disease cohort, our study offers a novel perspective and generates hypotheses for future investigations. Furthermore, given their non-invasive nature, reproducibility, and ease of incorporation into routine electrophysiological evaluations, MUNIX and MUSIX may also have potential utility as monitoring tools for longitudinal assessment of neuromuscular involvement in Fabry disease. Larger multicenter studies and integrated functional measures are warranted to validate these findings and further define the role of MUNIX in characterizing neuromuscular involvement in Fabry disease.

The following are the supplementary data related to this article.

**Supplementary Fig. S1:**
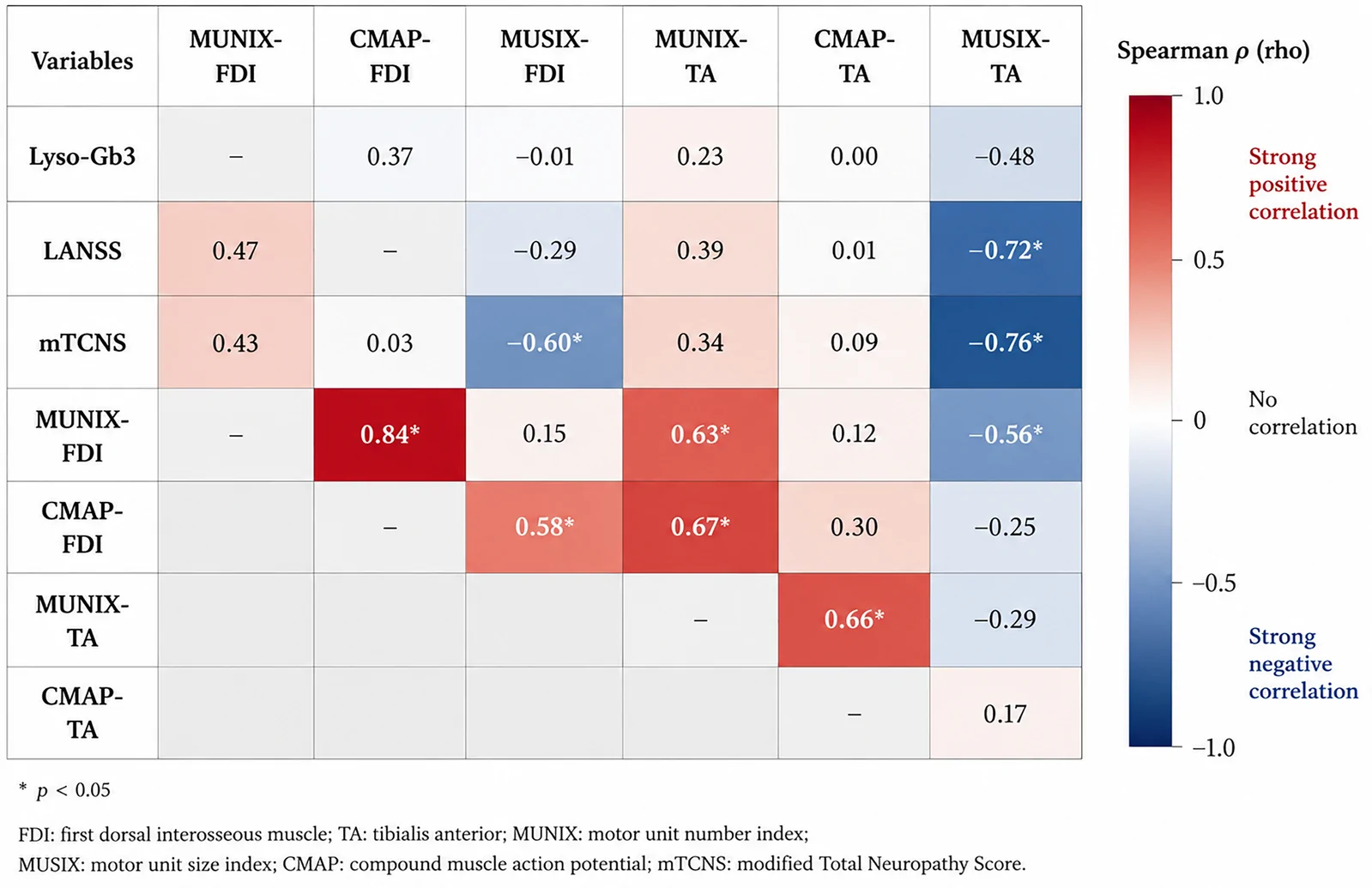
Heatmap of correlations between clinical, biochemical, and neurophysiological parameters. Heatmap illustrating Spearman correlation coefficients (ρ) between Lyso-Gb3, LANSS score, mTCNS score, and MUNIX-, CMAP-, and MUSIX-derived parameters obtained from the first dorsal interosseous (FDI) and tibialis anterior (TA) muscles. Positive correlations are shown in warmer colors, whereas negative correlations are shown in cooler colors. Correlation coefficients are displayed within each cell. Statistically significant correlations are indicated by black boxes and asterisks (*p < 0.05, **p < 0.01, ***p < 0.001). Blank cells indicate analyses that were not performed or are not applicable.

Supplementary material Heatmap of correlations between clinical, biochemical, and neurophysiological parameters.

## Declaration of competing interest

The authors declare that they have no known competing financial interests or personal relationships that could have appeared to influence the work reported in this paper.
