# Supplementary material for: Impaired motor unit profile in Fabry disease: A quantitative electrophysiological study
Source: Clin Neurophysiol Pract. 2026 Aug 25;11:749–56. doi: 10.1016/j.cnp.2026.08.010 (PMC13560524; doi:10.1016/j.cnp.2026.08.010)
Supplement: Supplementary file 1 [file mmc2.docx]

**Supplementary Table 1. Test–retest reproducibility of MUNIX and MUSIX measurements**

| **Muscle** | **Parameter** | **Pearson *r*** | ***p* value** | **Mean bias (Retest − Test)** | **95% LoA** | **CoV (%)** |
| --- | --- | --- | --- | --- | --- | --- |
| TA | MUNIX | 0.922 | <0.001 | −6.4 | −28.0 to 15.2 | 14.7 |
| TA | MUSIX | 0.843 | 0.002 | −5.6 | −26.7 to 15.5 | 13.3 |
| FDI | MUNIX | 0.894 | <0.001 | −34.8 | −84.4 to 14.7 | 20.7 |
| FDI | MUSIX | 0.912 | <0.001 | −11.7 | −30.5 to 7.2 | 16.4 |

*r*, Pearson correlation coefficient; LoA, limits of agreement; CoV, coefficient of variation. Bias represents the mean difference between retest and test measurements (retest − test).

**Supplementary Table 2. Spearman correlation coefficients (ρ) and p-values between biochemical, clinical, autonomic, and electrophysiological parameters in patients with Fabry disease**

| **Variables** | **MUNIX-FDI** | **CMAP-FDI** | **MUSIX-FDI** | **MUNIX-TA** | **CMAP-TA** | **MUSIX-TA** |
| --- | --- | --- | --- | --- | --- | --- |
| **Lyso-Gb3** | **ρ=0.55, p=0.049*** | ρ=0.37, p=0.209 | ρ=–0.01, p=0.964 | ρ=0.23, p=0.452 | ρ=0.00, p=0.993 | ρ=–0.48, p=0.097 |
| **LANSS** | ρ=0.47, p=0.146 | ρ=0.30, p=0.372 | ρ=–0.29, p=0.390 | ρ=0.39, p=0.240 | ρ=0.01, p=0.968 | **ρ=–0.72, p=0.013*** |
| **mTCNS** | ρ=0.43, p=0.164 | ρ=0.03, p=0.931 | **ρ=–0.60, p=0.037*** | ρ=0.34, p=0.282 | ρ=0.09, p=0.790 | **ρ=–0.76, p=0.004*** |
| **MUNIX-FDI** | **—** | **ρ=0.84, p<0.001*** | ρ=0.15, p=0.615 | **ρ=0.63, p=0.020*** | ρ=0.12, p=0.687 | **ρ=–0.56, p=0.044*** |
| **CMAP-FDI** | **—** | **—** | **ρ=0.58, p=0.038*** | **ρ=0.67, p=0.012*** | ρ=0.30, p=0.319 | ρ=–0.25, p=0.414 |
| **MUNIX-TA** | **—** | **—** | **—** | **—** | **ρ=0.66, p=0.014*** | ρ=–0.29, p=0.334 |
| **CMAP-TA** | **—** | **—** | **—** | **—** | **—** | ρ=0.17, p=0.579 |

*p < 0.05 statistically significant.

Abbreviations: **LANSS** – Leeds Assessment of Neuropathic Symptoms and Signs; **mTCNS** – modified Toronto Clinical Neuropathy Score; **MUNIX** – motor unit number index; **MUSIX** – motor unit size index; **CMAP** – compound muscle action potential; **FDI** – first dorsal interosseous muscle; **TA** – tibialis anterior muscle.
